# Different expression levels of interleukin-36 in asthma phenotypes

**DOI:** 10.1186/s13223-023-00868-2

**Published:** 2024-01-13

**Authors:** Jinyan Li, Zhengda Wang, Hongna Dong, Yuqiu Hao, Peng Gao, Wei Li

**Affiliations:** Department of Respiratory and Critical Care Medicine, The Second Hospital of Jilin University, Changchun, Jilin China

**Keywords:** Asthma, IL-36, Induced sputum, Asthma phenotypes, Asthma patients

## Abstract

Interleukin (IL)-36 family is closely associated with inflammation and consists of IL-36α, IL-36β, IL-36γ, and IL-36Ra. The role of IL-36 in the context of asthma and asthmatic phenotypes is not well characterized. We examined the sputum IL-36 levels in patients with different asthma phenotypes in order to unravel the mechanism of IL-36 in different asthma phenotypes. Our objective was to investigate the induced sputum IL-36α, IL-36β, IL-36γ, and IL-36Ra concentrations in patients with mild asthma, and to analyze the relationship of these markers with lung function and other cytokines in patients with different asthma phenotypes. Induced sputum samples were collected from patients with mild controlled asthma (n = 62, 27 males, age 54.77 ± 15.49) and healthy non-asthmatic controls (n = 16, 10 males, age 54.25 ± 14.60). Inflammatory cell counts in sputum were determined. The concentrations of IL-36 and other cytokines in the sputum supernatant were measured by ELISA and Cytometric Bead Array. This is the first study to report the differential expression of different isoforms of IL-36 in different asthma phenotypes. IL-36α and IL-36β concentrations were significantly higher in the asthma group (*P* = 0.003 and 0.031), while IL-36Ra concentrations were significantly lower (*P* < 0.001) compared to healthy non-asthmatic controls. Sputum IL-36α and IL-36β concentrations in the neutrophilic asthma group were significantly higher than those in paucigranulocytic asthma (n = 24) and eosinophilic asthma groups (n = 23). IL-36α and IL-36β showed positive correlation with sputum neutrophils and total cell count (R = 0.689, *P* < 0.01; R = 0.304, *P* = 0.008; R = 0.689, *P* < 0.042; R = 0.253, *P* = 0.026). In conclusion, IL-36α and IL-36β may contribute to asthma airway inflammation by promoting neutrophil recruitment in airways. Our study provides insights into the inflammatory pathways of neutrophilic asthma and identifies potential therapeutic target.

## Introduction

Asthma is a global bronchial inflammatory disease that affects individuals in all age-groups, and its prevalence has shown an increasing trend in many countries [1]. Asthma is a heterogeneous disease characterized clinically by reversible bronchoconstriction and airway hyperresponsiveness [2]. Asthma can be classified into 4 phenotypes based on the predominant type of inflammatory cells in the sputum: eosinophilic asthma (EA), neutrophilic asthma (NA), paucigranulocytic asthma (PA), and mixed granulocytic asthma (MA) [3]. Distinguishing the asthma phenotypes facilitates the analysis of clinical features, biological markers, and individualized treatment. NA is usually associated with more severe asthma, glucocorticoid resistance, and poor prognosis [3]. Therefore, identifying relevant biomarkers and developing therapeutic strategies for NA are key research imperatives.

Interleukin (IL-36) is a member of the IL-1 superfamily of three endogenous agonists, IL-36α, -β, and -γ, which promote inflammatory cell infiltration through signaling at the IL-36 receptor (IL-36R) [4]. Under physiological conditions, low levels of IL-36 cytokine expression can be observed in organs such as the skin, intestine, lung and brain; during inflammation, IL-36 receptor agonists are predominantly expressed by keratinocytes, epithelial cells, and inflammatory monocytes/macrophages [5]. IL-36 cytokines are activated by neutrophil-derived cathepsin G, elastase, and protease-3, which are mainly released by activated neutrophils [6]. Studies have indicated the potential involvement of IL-36 in a wide range of inflammatory and oncogenic processes in the skin, lung, kidney, liver, and intestine, which is mediated via activation of immune and non-immune cells, such as T cells, keratinocytes, and epithelial cells [7]. In a mouse model of unilateral ureteral obstruction, IL-36α was found to activate the IL-23/IL-17 axis, amplify inflammation, and promote the development of renal lesions. We hypothesized that a similar phenomenon may occur in the context of asthma [8]. A study found that IL-36γ promotes allergic rhinitis by enhancing eosinophil infiltration, and that IL-36α is involved in the allergic inflammatory response by regulating Th17 [9]. There are many similarities in the pathogenesis of allergic asthma and allergic rhinitis, and these are common diseases that frequently occur together [10].

As mentioned above, the heterogeneity of asthma and IL-36 may lead to inconsistency between the results of experimental studies. Therefore, in this study, we compared the sputum concentrations of IL-36 in asthma and healthy non-asthmatic individuals, and investigated the relationship between IL-36 and associated inflammatory cytokines. Furthermore, we investigated the sputum concentration of IL-36 in patients with different asthma phenotypes.

## Methods

### Study population

The diagnosis of asthma was based on the Global Initiative for Asthma (GINA) guidelines for current episodes of respiratory symptoms, evidence of variable airflow obstruction, and clinical diagnosis [11]. This study required sputum induction maneuvers; therefore, only asthmatic patients in a mild controlled stage were enrolled. The exclusion criteria were [1] pregnant women [2]; patients with severe cardiovascular diseases [3]; malignant tumors [4]; active tuberculosis or interstitial lung disease [5]; history of oral corticosteroid or antibiotic therapy in the past year [6]; exacerbation of asthma within the 4-week period immediately preceding the study; and [7] previous change of treatment within 4 weeks.

In addition, age- and sex-matched healthy non-asthmatic subjects were also recruited as healthy non-asthmatic controls. All asthma patients and healthy non-asthmatic subjects were recruited from the Second Hospital of Jilin University. All subjects completed a bronchodilator test prior to enrolment. All subjects were of Mongolian ethnicity, i.e., yellow race. All subjects completed the questionnaires, including treatment history, smoking history, and presence of respiratory symptoms. All subjects provided written informed consent. The Ethics Committee of the Second Hospital of Jilin University granted ethical approval for this study (2016-34).

### Sputum collection

All participants inhaled ultrasonically nebulized hypertonic saline (4.5%) for 15 min to induce sputum after adequate cleaning of the oral cavity and pharynx. The induced sputum was collected into petri dishes and the sputum plugs were isolated. Dithiothreitol (DTT) was added to lyse the sputum plugs and the volume of sputum plugs was recorded. After 30 min of rotational mixing at room temperature, phosphate buffer solution (PBS, pH 7.4) with 4 times the volume of sputum was added and mixed. The filtered filtrate (60 μm) was centrifuged at 400×*g* for 10 min and the supernatant was stored at − 80 °C for subsequent experiments. Sputum cell smears were prepared by cell precipitation, fixed in methanol for 10 min, rinsed, stained with hematoxylin for 30 s, rinsed with Chromotrope 2R (C2R acid)-paraffin mixture for 20 min, rinsed again, air-dried, and sealed with neutral resin [12, 13].

### Measurement of IL-36 and other cytokines

The concentrations of IL-36α, IL-36β, IL-36γ, IL-36Ra, and IL-1β were measured using a commercial human ELISA kit (CUSABIO, China). The IL-2, IL-4, IL-6, IL-9, IL-10, IL-13, IL-17 A, IL-17 F, IL-22, IFN-γ, and TNF-α concentrations were determined using the Multi-Analyte Flow Assay Kit (Biolegend, USA) with a Cytometric Bead Array (CBA). The above assay steps were performed according to the manufacturer’s recommended protocol.

### Asthma phenotype classification

The numbers of various inflammatory cells in the induced sputum smear were observed microscopically and recorded. Patients with neutrophils ≥ 61% in sputum were categorized as NA, patients with eosinophils ≥ 3% in sputum as EA, patients with eosinophils < 3% and neutrophils < 61% in sputum as PA, and patients with eosinophils ≥ 3% and neutrophils ≥ 61% in sputum were categorized as MA [14].

### Statistical analysis

All data were analyzed using the Statistical Package for the Social Sciences for Windows (SPSS) statistical software Version 20 (SPSS Inc., IL, USA). Non-normally distributed continuous variables were subjected to logarithmic transformation, after which statistical analysis was performed on normally distributed logged data. Normally distributed variables were expressed as mean ± standard deviation (SD) and statistical analysis was performed using ANOVA with a least significant difference (LSD). Non-normally distributed variables were expressed as median and interquartile range (IQR), and statistical analysis was performed using Kruskal Wallis H test with Bonferroni correction or Mann–Whitney U test. Categorical variables were analyzed using Chi-squared test. Correlations between each inflammatory factor in sputum supernatant, and correlation of inflammatory factors with lung function, and inflammatory cells in sputum were analyzed using partial correlation. *P* values < 0.05 were considered indicative of statistical significance.

## Results

### Clinical characteristics of asthmatic patients and healthy non-asthmatic controls

A total of 62 patients with asthma (27 male and 35 female) were included in this study. Sixteen healthy volunteers (10 males and 6 females) were enrolled in the control group. There were no significant between-group differences with respect to the baseline clinical data (*P* > 0.05). The predicted and post values of forced expiratory volume in 1 s (FEV1) in the asthma group were significantly lower than those in the healthy non-asthmatic control group (*P* < 0.001 and 0.002, respectively). The number of eosinophils, neutrophils, macrophages, and lymphocytes in the induced sputum were significantly greater in the asthma group compared to the control group (eosinophils: *P* < 0.001, neutrophils, macrophages, lymphocytes: *P* = 0.001) (Table 1).

**Table 1:** Characteristics of asthma patients and healthy non-asthmatic controls

Variable	Asthma	Normal	*P* value
Number	62	16	
Age, years	54.77 ± 15.49	54.25 ± 14.60	0.903
Sex, male, n (%)	27 (43.5)	10 (62.5)	0.179
BMI, kg/m^2^	24.10 ± 3.76	23.75 ± 3.45	0.632
Ex-smoker, n (%)	29 (46.8)	5 (31.3)	0.267
Pre-FEV1, L	1.78 ± 0.80	2.70 ± 0.78	< 0.001
Post-FEV1, L	2.06 ± 0.80	2.78 ± 0.80	0.002
Post-FVC, L	3.03 ± 0.81	3.45 ± 0.91	0.075
Post-bronchodilator FEV1/pred (%)	73.94 ± 23.96	97.68 ± 11.80	< 0.001
Post-bronchodilator FVC/pred (%)	89.90 ± 18.95	101.04 ± 12.48	0.029
Post-bronchodilator FEV1/FVC (%)	66.86 ± 14.57	80.16 ± 3.89	0.001
Sputum TCC, 10^6^/mL	0.97 (0.44,2.60)	1.65 (0.95,2.35)	0.319
Sputum NEU, 10^4^/mL	4.05 (0.58,49.65)	0.60 (0.23,0.93)	0.001
Sputum EOS, 10^4^/mL	2.95 (0.20,9.28)	0.00 (0.00,0.10)	< 0.001
Sputum MA, 10^6^/mL	71.45 (26.75,88.78)	98.15 (97.85,99.08)	< 0.001
Sputum LY, 10^4^/mL	6.00 (1.68,10.05)	0.65 (0.28,1.18)	< 0.001

Data are expressed as mean ± SD or median (IQR). Data were analyzed by Student’s *t* test or Mann–Whitney U test after adjusting for age

*BMI* body mass index, *FEV1* forced expiratory volume in 1 s, *FVC* forced vital capacity, *TCC* total cell count, *NEU* neutrophils, *EOS* eosinophils, *MA* Macrophages, *LY* lymphocytes

**Fig. 1:**
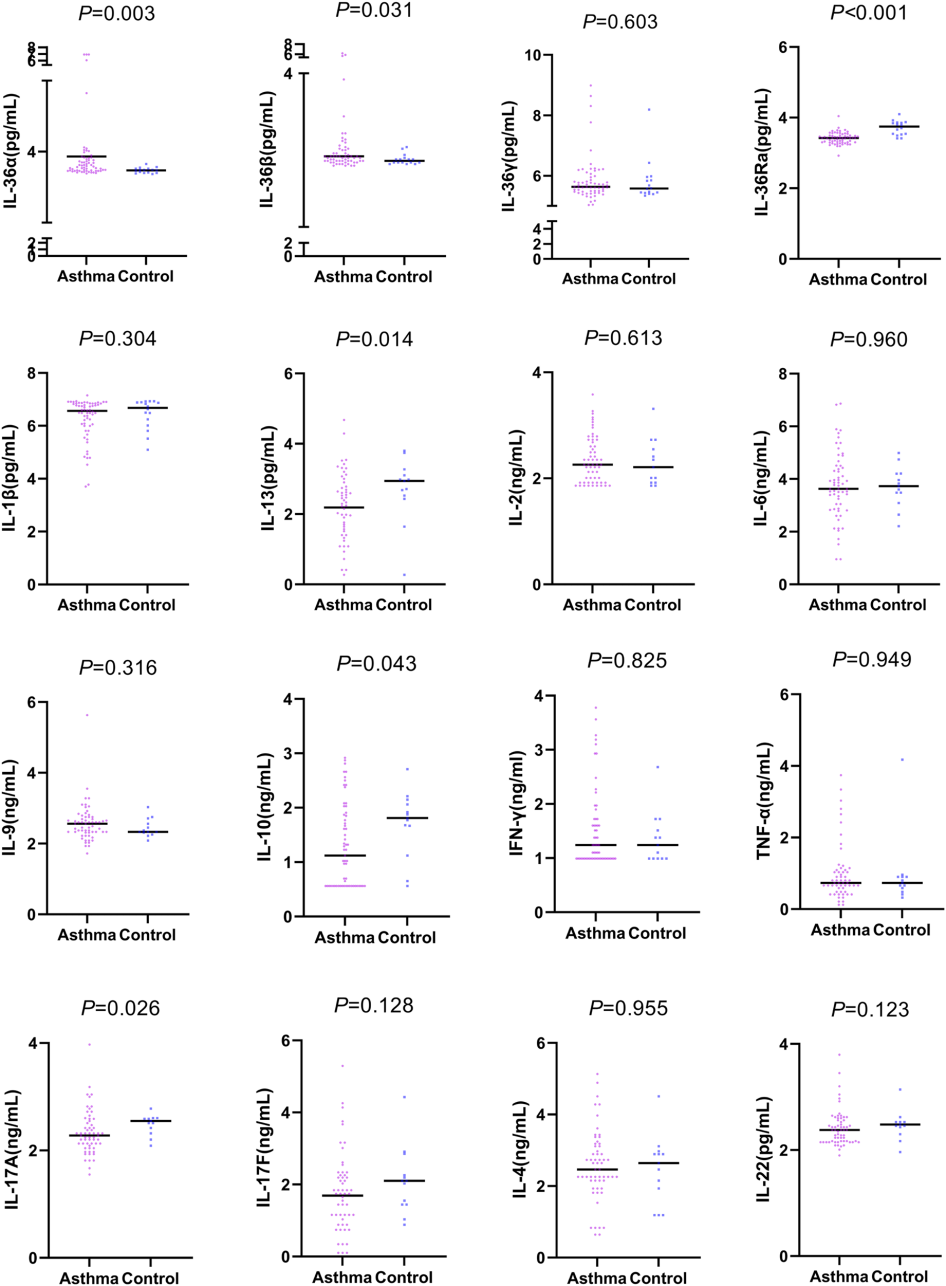
Relationship between inflammatory factors in asthma and healthy non-asthmatic controls. After logarithmic conversion and adjusting for age, the data are expressed as the individual geometric mean values and statistically analyzed. Horizontal lines represent the mean. *IL* interleukin, *TNF* tumor necrosis factor, *IFN* interferon

In the asthma group, the concentrations of IL-36α and IL-36β were significantly higher (*P* = 0.003 and 0.031), while the IL-36Ra concentration was significantly lower compared to the control group (*P* < 0.001). However, there was no significant between-group difference with respect to IL-36γ concentration (*P* = 0.603). The concentrations of IL-10, IL-13, and IL-17 A in the asthma group were significantly lower than those in the control group (IL-10: *P* = 0.043; IL-13: *P* = 0.014; IL-17 A: *P* = 0.026). There were no significant between-group differences with respect to the other inflammatory factors (Fig. 1).

### Clinical features of inflammatory phenotypes in asthma

**Fig. 2:**
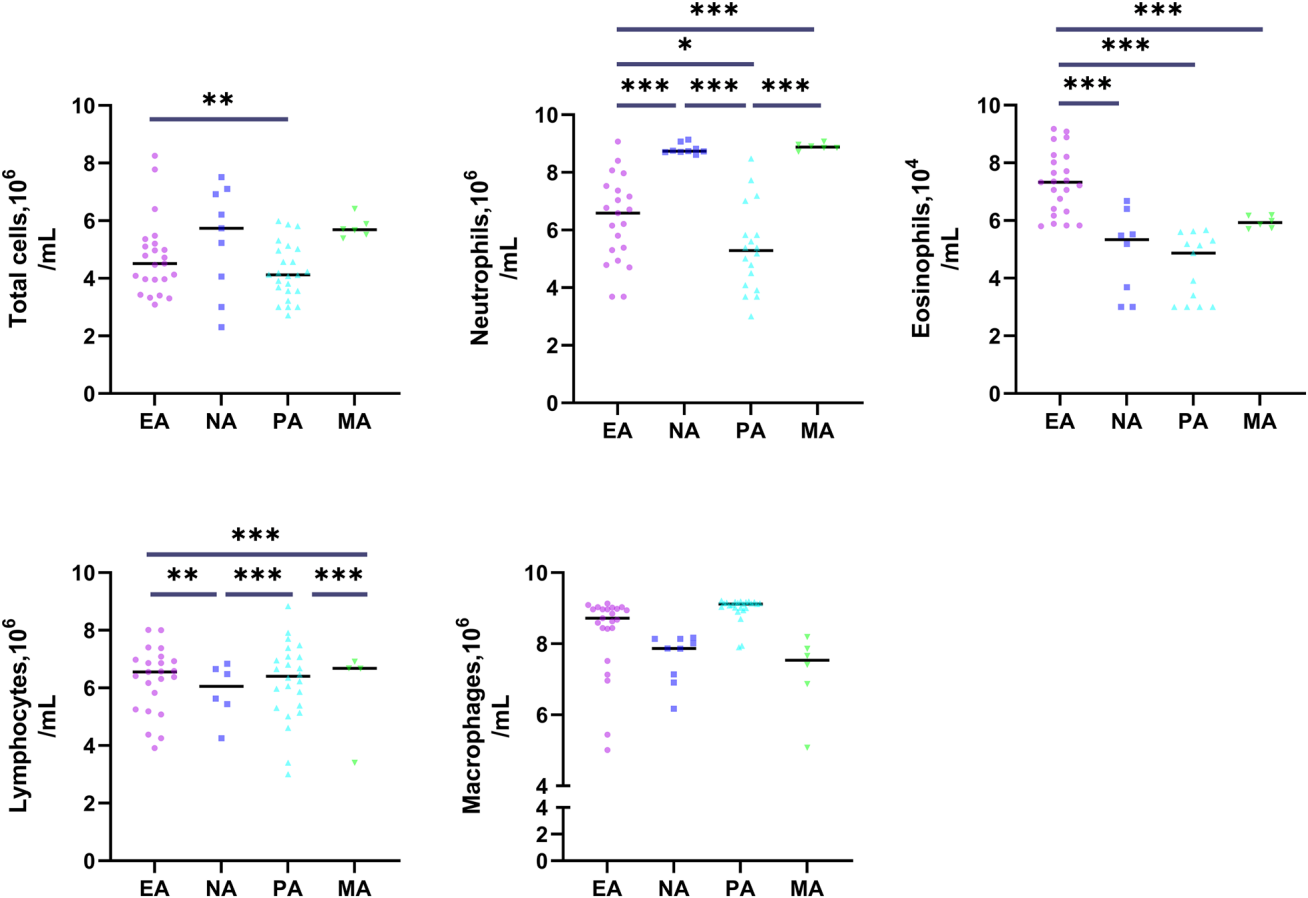
Sputum cell numbers in asthma in inflammatory phenotypes. After logarithmic conversion and adjusting for age, the data are expressed as the individual geometric mean values and statistically analyzed. Horizontal lines represent the mean values. *EA* eosinophilic asthma, *NA* neutrophilic asthma, *PA* paucigranulocytic asthma, *MA* mixed granulocytic asthma. *P < 0.05; **P < 0.01; ***P < 0.001

The asthma group was further divided into EA, MA, NA, and PA groups based on the examination of induced sputum; the clinical characteristics of these groups were comparable (*P* > 0.05) (Table 2 and Fig. 2).

**Table 2:** Clinical characteristics and sputum cell numbers in asthma inflammatory phenotypes

Variable	EA	NA	PA	MA	*P* value
Number	23	9	24	6	
Age, yrs	50.48 ± 17.33	59.44 ± 12.36	54.83 ± 14.80	64.00 ± 10.58	0.192
Male, n (%)	13 (56.5)	1 (11.1)	11 (45.8)	2 (33.3)	0.129
BMI, kg/m^2^	23.48 ± 3.99	24.89 ± 4.05	24.42 ± 3.62	24.00 ± 3.52	0.762
Ex-smoker, n (%)^a^	10 (43.5)	5 (55.6)	12 (50)	2 (33.3)	0.801
Pre-bronchodilator FEV1, L^a^	1.93 ± 0.92	1.53 ± 0.83	1.74 ± 0.73	1.78 ± 0.58	0.670
Post-bronchodilator FEV1, L^a^	2.21 ± 0.97	1.86 ± 0.84	1.98 ± 0.65	2.08 ± 0.61	0.644
Post-bronchodilator FVC, L^a^	3.20 ± 0.96	3.08 ± 0.96	2.87 ± 0.63	2.96 ± 0.65	0.616
Post-bronchodilator FEV1/pred (%)	72.53 ± 21.33	64.37 ± 25.94	77.16 ± 26.99	80.81 ± 17.17	0.495
Post-bronchodilator FVC/pred (%)	88.78 ± 14.25	85.24 ± 21.31	91.47 ± 22.21	94.90 ± 20.09	0.761
Post-bronchodilator FEV1/FVC (%)	67.14 ± 14.56	57.92 ± 13.68	68.90 ± 14.16	71.06 ± 15.74	0.227

The cells were tested in the sputum samples. Data are expressed as mean ± SD or median (IQR). Data were analyzed by ANOVA or Kruskal–Wallis test. Abbreviations as in Table 1

^a^Adjusted for age

### Concentrations of IL-36 and other inflammatory mediators in Asthma phenotypes

**Fig. 3:**
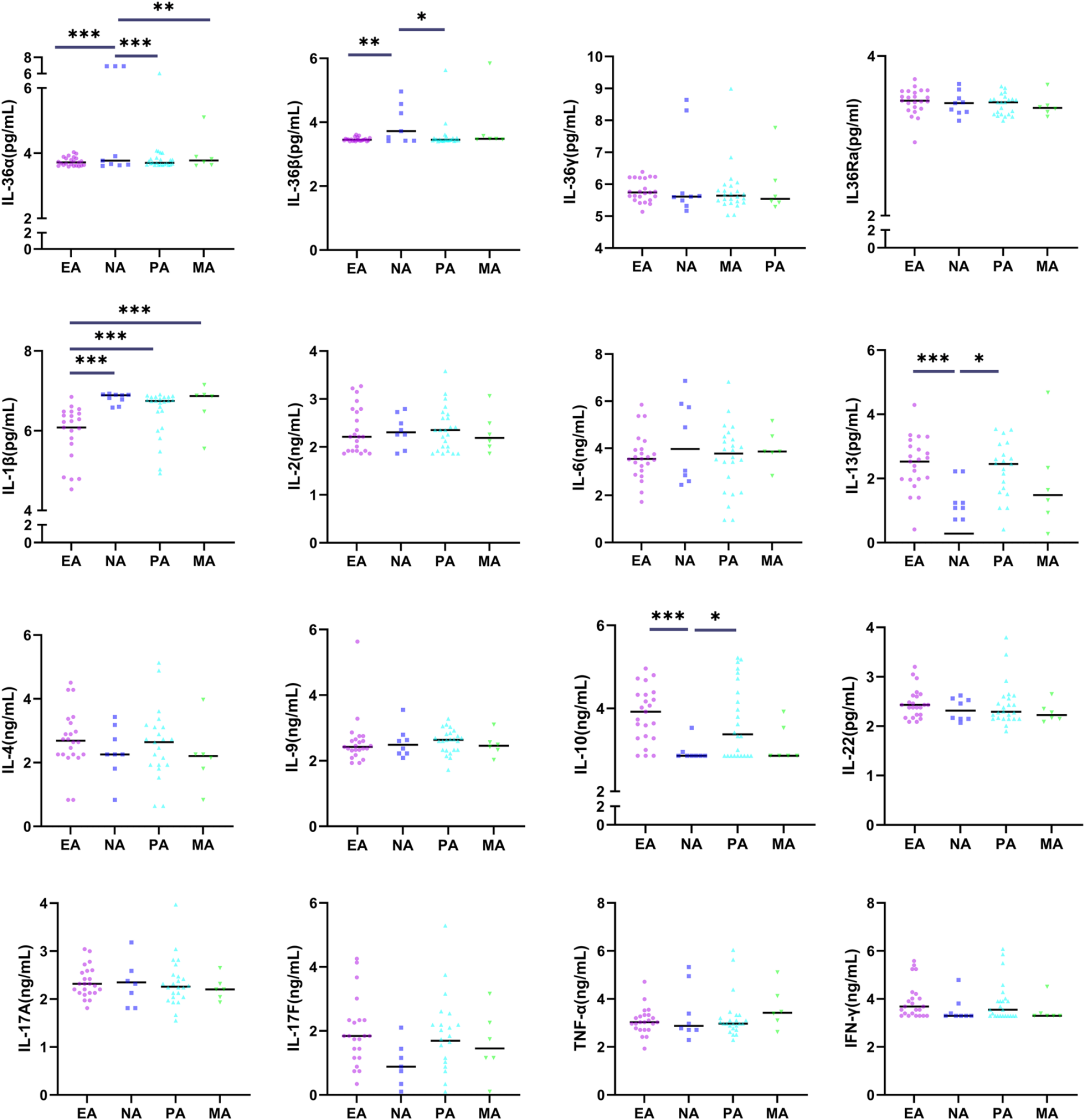
Concentrations of inflammatory mediators in sputum supernatant of different subtypes of asthma patients. After logarithmic conversion and adjusting for age, the data are expressed as the individual geometric mean values and statistically analyzed. Horizontal lines represent the geometric mean. Abbreviations as in Fig. 1. *P < 0.05; **P < 0.01; ***P < 0.001

Sputum IL-36α and IL-36β concentrations in the NA group were significantly higher than those in the PA and EA groups. Sputum IL-1β concentration in the NA, PA, and MA groups were significantly higher than that in the EA group. Sputum IL-13 and IL-10 concentrations in the NA group were significantly lower than those in the PA and EA groups. Sputum IL-6 concentration in the NA group was significantly higher than that in the EA group. The concentrations of other inflammatory factors were comparable among the groups (Fig. 3).

### Association between IL-36 and inflammatory cells

**Fig. 4:**
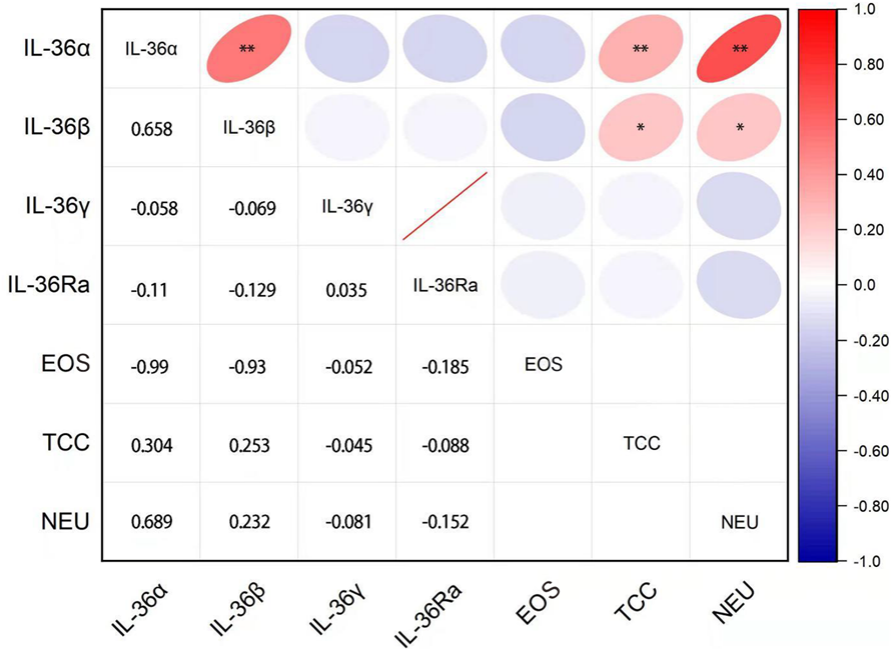
Correlation of IL-36 and cellular levels in sputum supernatant of asthma patients. The data are expressed as individual values and were analyzed by partial correlation after adjusting for age and sex. *TCC* total cell count, *NEU* neutrophils, *EOS* eosinophils, *IL* interleukin. *P < 0.05; **P < 0.01

We compared inflammatory mediators and concentrations of inflammatory cells in the induced sputum. IL-36α and IL-36β showed positive correlation with sputum neutrophils and total cell count (TCC) (R = 0.689, *P* < 0.01; R = 0.304, *P* = 0.008; R = 0.689, *P* < 0.042; R = 0.253, *P* = 0.026). In addition, there was a significant positive correlation between IL-36α and IL-36β (R = 0.658, *P* < 0.01) (Fig. 4).

### Association of IL-36 with other inflammatory mediators

We compared the concentrations of IL-36 and other inflammatory mediators in the sputum supernatant. IL-36α, IL-36β, and IL-36γ showed strong positive correlation with IL-6, TNF-α, and IL-17 A, respectively (R = 0.592, 0.451, and 0.431, *P* < 0.01) (Table 3).

**Table 3:** IL-36 and sputum chemokine correlations

Variable	IL-36α, pg/mL		IL-36β, pg/mL		IL-36γ, pg/mL		IL-36Ra, pg/mL	
	R	*P* value	R	*P* value	R	*P* value	R	*P* value
IL-1β, pg/mL	0.148	0.201	0.071	0.543	0.02	0.864	0.015	0.899
IL-2, ng/mL	0.066	0.584	− 0.004	0.971	− 0.068	0.570	0.073	0.544
IL-4, ng/mL	− 0.47	0.696	− 0.062	0.606	− 0.066	0.580	− 0.010	0.933
IL-6, ng/mL	0.592	< 0.01	0.207	0.082	− 0.82	0.494	− 0.088	0.464
IL-9, ng/mL	0.043	0.721	0.051	0.672	− 0.22	0.855	− 0.012	0.918
IL-10, ng/mL	− 0.166	0.163	− 0.203	0.088	− 0.107	0.373	0.022	0.852
IL-13, pg/mL	− 0.159	0.181	− 0.182	0.126	− 0.108	0.366	0.057	0.633
IL-17 A, ng/mL	− 0.017	0.889	− 0.033	0.785	0.431	< 0.01	0.078	0.515
IL-17 F, ng/mL	− 0.087	0.465	− 0.097	0.417	− 0.065	0.587	− 0.006	0.958
IL-22, pg/mL	− 0.002	0.988	0.086	0.473	− 0.027	0.822	0.213	0.073
IFN-γ, ng/mL	− 0.061	0.608	0.048	0.691	0.019	0.873	− 0.035	0.770
TNF-α, ng/mL	0.249	0.035	0.451	< 0.01	0.219	0.065	0.028	0.818

Data were analyzed by partial correlation after adjusting for age and BMI

*IL* interleukin, *TNF* tumor necrosis factor, *IFN* interferon

### Association of other inflammatory mediators

**Fig. 5:**
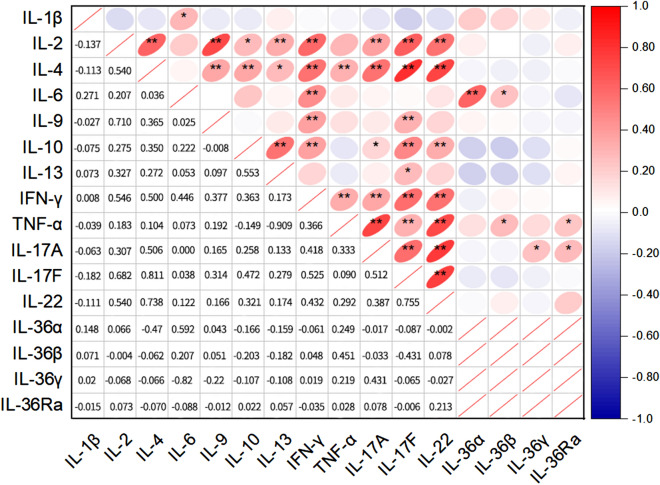
Correlation analysis of other inflammatory factors. Data are expressed as individual values and were analyzed by partial correlation after adjusting for age. Abbreviations as in Fig. 1. *P < 0.05; **P < 0.01

In addition, our study also innovatively performed multiple comparisons of IL-2, IL-4, IL-6, IL-9, IL-10, IL-13, IL-17 A, IL-17 F, IL-22, IFN-γ, and TNF-α (Fig. 5). Our results found a significant positive correlation between IL-2 (R = 0.614) and IL-4 (R = 0.614), IL-9 (R = 0.710), IL-10 (R = 0.275), IL-13 (R = 0.327), IL-17 A (R = 0.307), IL-17 F (R = 0.628), IL-22 (R = 0.540), IFN-γ (R = 0.546) (*P* < 0.05). IL-1β had a significant positive correlation with IL-6 (R = 0.271; *P* < 0.05). IL-13 had a significant positive correlation with IL-2 (R = 0.327), IL-4 (R = 0.272), IL-10 (R = 0.553), IL-17 F (R = 0.279) (*P* < 0.05). IL-4 had a significant positive correlation with IL-2 (R = 0.614), IL-9 (R=,0.365), IL-10 (R = 0.350), IL-13 (R = 0.272), IL-17 A (R = 0.506), IL-17 F (R = 0.811), IL-22 (R = 0.738), IFN-γ (R = 0.500) (*P* < 0.05). IL-6 was significantly and positively correlated with IL-1β, IFN-γ (R = 0.271, 0.446; *P* < 0.05). IL-9 had significant positive correlation with IL-2 (R = 0.710), IL-4 (R = 0.365), IFN-γ (R = 0.377), IL-17 F (R = 0.314) (*P* < 0.05). IL-10 had significant positive correlation with IL-2 (R = 0.275), IL-13 (R = 0.553), IFN-γ (R = 0.363), IL-17 F (R = 0.258) (*P* < 0.05). iFN-γ was significantly correlated with IL-2 (R = 0.546), IL-4 (R = 0.500), IL-6 (R = 0.446), IL-9 (R = 0.377), IL-10 (R = 0.363), IL-17 A (R = 0.418), IL-17 F (R = 0.525), IL-22 (R = 0.432), and TNF-α (R = 0.366) (*P* < 0.05). TNF-α had significant positive correlation with IL-17 A, IL-22, and IFN-γ (R = 0.333, 0.292, 0.366; *P* < 0.05). IL-17 A had significant positive correlation with IL-2 (R = 0.307), IL-4 (R = 0.506), IL-10 (R = 0.258), IL-17 F (R = 0.512), IL-22 (R = 0.378), IFN-γ (R = 0.418), and TNF-α (R = 0.333) (*P* < 0.05). IL-17 F showed a significant positive correlation with IL-2 (R = 0.628), IL-4 (R = 0.811), IL-9 (R = 0.314), IL-10 (R = 0.472), IL-13 (R = 0.279), IL-17 A (R = 0.512), IL-22(R = 0.755), IFN-γ(R = 0.525) (*P* < 0.05). IL-22 was significantly correlated with IL-2 (R = 0.540), IL-4 (R = 0.738), IL-10 (R = 0.321), IFN-γ (R = 0.432), TNF-α (R = 0.292), IL-17 A (R = 0.378), and IL-17 F (R = 0.755) (*P* < 0.05).

## Discussion

The involvement of IL-36 in the pathogenesis of autoimmune diseases is well established. However, its role in the pathogenesis of asthma is not well characterized. IL-Rrp2 is the common binding receptor for all IL-36 isoforms, and IL-36α, IL-36β, and IL-36γ compete with IL-36Ra for binding to this receptor [15]. In our study, asthmatic patients had higher sputum IL-36α and IL-36β concentrations, and lower IL-36Ra concentration compared to healthy non-asthmatic controls. In a mouse model of *S. aureus*-induced epidermal inflammation, IL 36α and IL-4 released from keratinocytes were found to promote B-cell IgE secretion, plasma cell differentiation, and elevated serum IgE concentrations. However, these changes were significantly attenuated in IL-36R-deficient transgenic mice or wild-type mice treated with anti-IL-36R antagonistic antibodies [16]. Our results support this study; however, there is a paucity of studies on IL-36 isoforms in different asthmatic phenotypes. Therefore, we sought to investigate whether IL-36 concentrations differed among asthma phenotypes, and if so, whether these differences could be explained by heterogeneity of asthma inflammation or differences in asthma phenotypes. We further examined the concentrations of various IL-36 subtypes in the sputum supernatant of patients with different asthmatic phenotypes.

Interestingly, sputum IL-36α and IL-36β concentrations were significantly higher in the NA group compared to the PA and EA groups. However, there were no significant differences between the phenotypes with respect to sputum IL-36γ and IL-36Ra. Moreover, IL-36α and IL-36β showed a positive correlation with sputum neutrophils and TCC. These findings indicate a key role of IL-36 isoforms in inducing infiltration and activity of neutrophils in asthma, and underline their involvement in the pathophysiology of airway inflammation in the asthmatic phenotypes. IL-36α has a pro-inflammatory effect on the lung. One study found that the neutrophil environment can activate IL-36α and IL-36γ [17]. Intratracheal administration of IL-36α drops in a mouse model was found to induce the activation of the NF-κB and MAPK pathways, and induce neutrophil chemokine expression, ultimately leading to neutrophil intracellular flow [18, 19]. In addition, IL-36 pro-inflammatory factors can promote the expression of neutrophil chemokines such as CXCL8, CXCL1, and CXCL2, which induce neutrophil endocytosis [19, 20]. IL-36 induces the production of pro-inflammatory factors such as IL-1β, TNF-α, IL-12, and IL-23. IL-36β is involved not only in inducing Th1 cell polarization but also in the Th1 immune response following mycobacterial infection [21]. These studies are consistent with our findings. In addition, IL-36 cytokines have been shown to be mainly involved in the Th1 immune response, while the in vivo expression of IL-36α and IL-36β promotes neutrophil recruitment in asthmatic airways [22, 23]. IL-36R expression is increased in naive CD4+ T cells, and IL-36β, together with IL-12, promotes the Th1 polarization of naive CD4+ T cells [21]. IL-36 has now been shown to be involved in the polarization process of Th17 [22]. IL-36α and IL-17 have a strong feedback loop in switching skin inflammation signaling [24]. Our study also found a significant positive correlation between IL-36γ and IL-17 A concentrations. It has been found that the level of IL-36γ increases after IL-17 stimulation [9]. Perhaps IL-36γ and IL-36β are jointly involved in the enhanced feedback loop of IL-17 for activating the immune response in asthma. IL-36α, IL-36β, IL-36γ, and IL-36Ra may be involved in the pathogenesis of asthma phenotypes via different pathways and may be important biological targets for asthma therapy. Our study also had an interesting finding. It is well known that IL-13 H and IL-17 are classical pro-inflammatory cytokines, usually expressed at higher levels in asthma patients. However, in our study, IL-13 and IL-17 levels were lower in the asthma group. This contradictory result is the reason for the further differentiation of asthma into four different subtypes in our study. The heterogeneity of asthma leads to such contradictory results; therefore, further studies to differentiate asthma into subtypes are important for individualized and precise treatment of asthma. In our study, we found that IL-13 and IL-10 levels were lower in neutrophilic asthma. IL-13 is a cytokine secreted mainly by Th2, typically accompanying Th2 asthma, and IL-13 correlates with the severity of asthma, including eosinophilic airway inflammation, mucus secretion, airway hyperresponsiveness, and remodeling. In addition, anti-IL-13 therapy plays a significant role in targeted asthma therapy. CCL11 (eotaxin1) and CCL17 promote eosinophil and leukocyte infiltration into the lung mediated by IL-13 [25–28]. One study found significantly increased IL-13 in BALF, lung block biopsy specimens, and sputum of asthmatics; however, further differentiation of asthma subtypes revealed that IL-13 was not increased in non-eosinophilic asthma [28, 29]. IL-10 is a cytokine with both anti-inflammatory and pro-inflammatory effects and is mainly produced by activated monocytes, peripheral blood T cells, B lymphocytes, macrophages, mast cells, eosinophils, and dendritic cells. In asthma, IL-10 can negatively regulate the inflammatory response mediated by Th2 and Th17 and can alleviate the severity of neutrophilic asthma [30]. Due to the complex function of IL-36, the results of different studies may not be consistent with each other. A previous study found significantly increased expressions of serum IL-36 cytokine mRNA and protein in patients with allergic rhinitis and asthma [31, 32], which is consistent with our study. However, the serum IL-36γ and IL-36R mRNA and protein expressions were also significantly elevated in patients with allergic rhinitis, which is different from our findings. These inconsistent findings may be attributable to the different proportions of patients with different asthma phenotypes in the study sample. In addition, our study also innovatively performed multiple comparisons of IL-2, IL-4, IL-6, IL-9, IL-10, IL-13, IL-17 A, IL-17 F, IL-22, IFN-γ, and TNF-α (Fig. 4). The cytokines that are closely related to IL-36 isoforms are described below. IL-1β plays a pro-inflammatory role in the pathogenesis of asthma. IL-1β expression was found in lavage fluid, epithelial cells, and alveolar macrophages of asthmatic patients. IL-1β is a regulator of airway hyperresponsiveness in asthma and can mediate eosinophil inflammation by inducing chemokines and cytokines. In addition, IL-1β is also involved in neutrophil-mediated inflammation [33]. IL-1β can promote the production of IL-6 and chemokines in the lung, recruit neutrophils, and promote the inflammatory response [34]. In addition, the pathogenesis of neutrophilic asthma is associated with IL-1β/IL-17-induced neutrophil activation [35, 36].

We determined that the pro-inflammatory factor IL-36 can promote neutrophil aggregation in asthma airway inflammation, but the exact underlying mechanisms are not clear [22]. Therefore, we further examined asthma-associated inflammatory factors and assessed their correlation with IL-36. We observed that IL-36α was positively correlated with IL-6; IL-36β was positively correlated with TNF-α, and IL-36γ was positively correlated with IL-17 A. IL-6 is known to induce neutrophil recruitment and its level increases with increasing neutrophil numbers [37]. IL-36α was shown to induce the composition of MyD88 linked molecules to form complexes and induce activation of JNK, MAPK, and ERK1/2 signaling pathways to enhance IL-6 expression [38]. Studies have shown that in the airway epithelium, IL-36α and IL-36γ promote IL-1β, IL-17 A, and TNF-α, an effect that is mediated through Toll-like receptors 2/6, 3, 4, and 5. This is consistent with our findings [39]. We also innovatively found a positive correlation between IL-36β and TNF-α, which was not found in previous experiments. In vitro, treatment of cultured human keratinocytes with TNF-α and IL-17 A resulted in significantly higher levels of IL-36α and IL-36γ, forming a positive feedback loop with Th17 cytokines, which also stimulated the production of pro-inflammatory cytokines such as TNF-α, IL-6, and IL-8 [40]. TNF-α is produced by a variety of pro-inflammatory cells and structural cells during the pathogenesis of asthma, and TNF-α is mainly associated with the Th1 response. It also works with IL-17 A to produce cxcl8, which promotes neutrophil aggregation, and is associated with the inflammatory mechanisms and airway hyperresponsiveness in neutrophilic asthma [41–44]. It plays an important role in airway remodeling and inflammatory response and promoting neutrophil and eosinophil migration by promoting pro-inflammatory factors and adhesion molecules such as vascular cell adhesion molecule 1 and intercellular adhesion molecule 1 [45]. Our study supports these results in that IL-36β showed a positive correlation with TNF-α. IL-17 A is a characteristic cytokine of TH17. A previous study described the association of IL-36 with TH17 cellular responses. In our study, IL-36γ showed a positive correlation with IL-17 A concentration, supporting our previously mentioned point. However, our study also found no significant differences in TNF-α and IL-17 A concentrations between the asthma group and healthy non-asthmatic controls, and between the different asthma phenotypes. This may be related to our sample size and the regional characteristics of asthma patients, and further underlines the heterogeneity of asthma. TNF-α, IL-17 A, and IL-1β act in consort with IL-36 to regulate Th1 cell responses by sharing downstream signals through pathways such as JNK, MAPK, ERK, p38, and NF-κB [24, 39]. IL-36 may promote airway neutrophil aggregation and airway inflammation through the IL-6/IL-17 A/TNF-α axis. Further exploration of the role of IL-36 receptor blockers in animal models of asthma and in vitro experiments are required to better characterize the role of IL-36 in asthma.

Based on our results, we suggest that IL-36 is associated with neutrophil recruitment in the airways and that IL-36 exacerbates the asthmatic airway inflammatory response via Th1-related cytokines. These results may serve as a basis for further investigation of the different pathophysiological mechanisms of IL-36 in NA and EA in the future.

## Conclusions

Our study indicates the involvement of IL-36α and IL-36β in the pathophysiology of airway inflammation in asthma, which is likely mediated via promotion of neutrophil recruitment in the airways. Our findings provide insights into the inflammatory pathways of neutrophilic asthma and identify a potential therapeutic target for the asthma phenotypes. However, more in vivo and in vitro experiments are required to investigate the role of IL-36 in various asthma phenotypes to assess the potential of IL-36-based therapeutic targets in asthma.

## Data Availability

All data generated or analyzed during this study are included in this article.

## Abbreviations

IL: Interleukin
TNF: Tumor necrosis factor
ELISA: Enzyme-linked immunosorbent assay
BMI: Body mass index
AHR: Airway hyperresponsiveness
ACQ: Asthma control questionnaire
FeNO: Fractional exhaled nitric oxide
FEV1: Forced expiratory volume in 1 s
FVC: Forced vital capacity
TCC: Total cell count
SD: Standard deviation
ANOVA: Analysis of variance
LSD: Least significant difference
IQR: Interquartile range
OR: Odd ratio
CI: Confidence interval
ICS: Inhaled corticosteroid
NA: Neutrophilic asthma
EA: Eosinophilic asthma
PA: Paucigranulocytic asthma
MGA: Mixed granulocytic asthma
